# Evaluation of recruitment and retention strategies for health workers in rural Zambia

**DOI:** 10.1186/1478-4491-12-S1-S1

**Published:** 2014-05-12

**Authors:** Fastone M Goma, Gail Tomblin Murphy, Adrian MacKenzie, Miriam Libetwa, Selestine H Nzala, Clara Mbwili-Muleya, Janet Rigby, Amy Gough

**Affiliations:** 1 School of Medicine, University of Zambia, Lusaka, Zambia; 2 WHO/PAHO Collaborating Centre on Health Workforce Planning and Research, Dalhousie University, Halifax, Canada; 3 HRH Directorate, Zambia Ministry of Health, Lusaka, Zambia; 4 Health Planning and Development, Lusaka District Health Management Team, Ministry of Health, Lusaka, Zambia

**Keywords:** Human resources for health, recruitment, retention, outcome mapping, Ressources humaines en santé, recrutement, maintien en poste, cartographie des incidences

## Abstract

**Background:**

In response to Zambia’s critical human resources for health challenges, a number of strategies have been implemented to recruit and retain health workers in rural and remote areas. Prior to this study, the effectiveness of these strategies had not been investigated. The purpose of this study was to determine the impacts of the various health worker retention strategies on health workers in two rural districts of Zambia.

**Methods:**

Using a modified outcome mapping approach, cross-sectional qualitative and quantitative data were collected from health workers and other stakeholders through focus group discussions and individual interview questionnaires and were supplemented by administrative data. Key themes emerging from qualitative data were identified from transcripts using thematic analysis. Quantitative data were analyzed descriptively as well as by regression modelling. In the latter, the degree to which variation in health workers’ self-reported job satisfaction, likelihood of leaving, and frequency of considering leaving, were modelled as functions of participation in each of several retention strategies while controlling for age, gender, profession, and district.

**Results:**

Nineteen health worker recruitment and retention strategies were identified and 45 health care workers interviewed in the two districts; participation in each strategy varied from 0% to 80% of study participants. Although a salary top-up for health workers in rural areas was identified as the most effective incentive, almost none of the recruitment and retention strategies were significant predictors of health workers’ job satisfaction, likelihood of leaving, or frequency of considering leaving, which were in large part explained by individual characteristics such as age, gender, and profession. These quantitative findings were consistent with the qualitative data, which indicated that existing strategies fail to address major problems identified by health workers in these districts, such as poor living and working conditions.

**Conclusions:**

Although somewhat limited by a small sample size and the cross-sectional nature of the primary data available, the results nonetheless show that the many health worker recruitment and retention strategies implemented in rural Zambia appear to have little or no impact on keeping health workers in rural areas, and highlight key issues for future recruitment and retention efforts.

## Abstract (fr)

En réponse aux problèmes de pénuries des ressources humaines dans le domaine de la santé en Zambie, un certain nombre de stratégies ont été mises en oeuvre pour recruter et maintenir en poste les travailleurs de la santé dans les régions rurales et éloignées. Avant cette étude, l’efficacité de ces stratégies n’avait pas été étudiée. L’objectif de cette étude était donc de déterminer l’incidence de différentes stratégies de maintien en poste de différents corps de métiers de la santé dans deux districts ruraux de la Zambie.

**Méthodes**

Au moyen d’une cartographie des incidences modifiée, des données transversales qualitatives et quantitatives ont été recueillies auprès des travailleurs de la santé et d’autres intervenants grâce à des groupes de discussion et des questionnaires individuels auxquels ont été intégrés des données administratives. Les principaux thèmes ont été dégagés des données qualitatives grâce à la transcription des discussions au moyen de l’analyse thématique. Les données quantitatives ont été analysées de façon descriptive ainsi qu’à l’aide d’un modèle de régression. Pour ce dernier, le degré de variation de la satisfaction par rapport à l’emploi, de la probabilité de quitter l’emploi et de la fréquence à laquelle les travailleurs pensaient à quitter leur emploi a été modelé en fonction de leur participation à chacune des différentes stratégies de maintien en poste, tout en considérant des données sur l’âge, le sexe, la profession et le district.

**Résultats**

Pas moins de 19 stratégies de recrutement et de maintien en poste ont été dénombrées auprès des 45 travailleurs de la santé interviewés dans les deux districts. La participation à chacune des stratégies variait de 0 à 80 % selon les participants à l’étude. Bien que les suppléments de rémunération pour les travailleurs de la santé dans les régions rurales constituent les mesures incitatives les plus efficaces, presque aucune stratégie de recrutement et de maintien en poste n’était un bon indicateur de la satisfaction face à l’emploi, de la probabilité de le quitter et de la fréquence à laquelle les travailleurs pensaient à quitter leur emploi. Ces facteurs étaient, dans une large part, expliqués par les caractéristiques des travailleurs comme l’âge, le sexe et la profession. Ces résultats quantitatifs étaient conformes aux données qualitatives, qui indiquaient que les stratégies de recrutement et de maintien en poste ne permettaient pas de régler les principaux problèmes déterminés par les travailleurs de la santé de ces districts, notamment des conditions de vie et de travail difficiles.

Malgré la taille de l’échantillon limitée et la nature transversale des données primaires disponibles, les résultats montrent néanmoins que les nombreuses stratégies de recrutement et de maintien en poste mises en oeuvre en Zambie rurale n’ont que peu ou pas du tout d’incidence sur le maintien en poste des travailleurs de la santé dans les régions rurales et mettent en lumière des problèmes de recrutement et de maintien en poste clés.

## Background

Zambia’s population faces many health-related challenges. For example, most of its citizens live in poverty, more than half its children suffer from chronic malnutrition, child mortality is greater than one in nine, and average life expectancy is only about 55 years [1]. Zambia is also burdened with rates of malaria [1,2] and HIV prevalence (12%) [1,2] among the highest in the world. Exacerbating these challenges and limiting the country’s ability to respond to them is its critical shortage of health workers [3,4].

Not only is there an absolute shortage of health workers in Zambia, the available workforce is unevenly distributed within the system [5]; the majority of health care providers work in urban centres while the majority of Zambia’s population live in rural areas [6]. Both absolute and relative shortages persist despite the application of innovative approaches to deal with human resources for health (HRH) issues [7]. Attrition rates among health workers are high as a result of migration to other countries and death due to HIV/AIDS, resulting in low staff motivation and poor morale [8-10]. In addition, there is significant internal migration from the public sector to those with better working conditions such as the private sector and non-governmental organizations (NGOs), and from rural areas to urban areas [11,12]. The shortage of trained health workers in rural and remote areas has deprived the majority of Zambians the “equity of access to quality health care as close to the family as possible” as stated in the country’s fifth Ministry of Health mission statement [13].

It was for the above reasons that the Zambia Ministry of Health (MoH) introduced a number of recruitment and retention strategies for Zambia’s public sector health workers in rural and remote parts of the country. Two of these are designed to compensate these health workers for the additional costs associated with living in rural and remote areas, namely rural and remote hardship allowances. Others are meant to compensate for the additional work hours often required of health workers in rural areas, including the on-call allowance (for doctors), night duty allowance, and commuted overtime allowance (for other professions). Other financial incentives include a retention allowance for university graduates, a uniform maintenance allowance (for nurses), an allowance for housing, and a salary top-up from the Churches Health Association of Zambia (CHAZ), a religious organization, for those working in their institutions. Non-financial incentives are designed to ensure health workers have access to basic infrastructure in their homes and workplaces. These include provision for housing rehabilitation, radio communication equipment (much of rural Zambia does not have cell phone coverage), motorcycles (many of Zambia’s rural areas are not accessible with a conventional automobile), solar panels (Zambia’s electrical network does not extend to all parts of the country), and well-drilling to ensure access to water. The most recent of these, the Zambia Health Workers’ Retention Scheme (ZHWRS) was launched in 2003 and initially targeted medical doctors; it was later scaled up to include other cadres of health professionals. The scheme originally included five components – a salary top-up, a child education allowance, housing rehabilitation, a car loan, and prioritized access to professional development opportunities.

Initial funding for the ZHWRS was approximately €2.3 million for the first three years, or €8,000 to €9,000 per participant per year [20]. More recently, the costs of the ZHWRS are estimated at about €4.4 million per year, or €4,380 per participant per year [personal communication with ZHWRS administrator]. This compares with an annual Ministry of Health budget of approximately €530 million (ZMK 3.63 billion) [21]. Costs associated with the various other schemes, which are administered differently from the ZHWRS, are not readily available.

The objective of this study was to measure the impacts of the various health worker recruitment and retention strategies on the health workforces of two rural districts in Zambia, Gwembe and Chibombo^i^. The specific research aims of the research were 1) to identify the formal strategies that had been implemented to recruit and retain health workers in Gwembe and Chibombo; and 2) to define the degree to which health workers’ participation in these strategies was associated with their job satisfaction and intention to stay in their jobs. Only members of health cadres targeted by the recruitment and retention strategies (i.e. excluding community health workers and support staff such as cleaners) were included in the study.

## Methods

The various recruitment and retention strategies implemented in the districts were identified through a combination of review of MoH reports and consultation with MoH personnel as well as with community representatives from each of the districts. Based on the scope and objectives of each scheme, indicators of their success were developed using an outcome mapping approach [14]. Health workers’ levels of participation in each scheme were identified as the key process indicators, while key outcome indicators were identified as participants’ satisfaction with each initiative, job satisfaction, and intention to stay or leave their jobs. Turnover or net health workforce growth rates at the district level were also a key outcome indicator.

A set of instruments to measure these indicators was developed. These included:

• An interview questionnaire for health workers to capture individual health worker characteristics such as age group, gender, and profession, as well as participation in each scheme, job satisfaction, the strategies rated as most effective, the likelihood of leaving their jobs, and the frequency with which they consider leaving their jobs;

• Questionnaires for district managers (in Gwembe and Chibombo), provincial health offices (South and Central, respectively), and the Ministry of Health, to collect administrative data from multiple sources on flows in and out of the health workforces in each district, as well as participation levels in each scheme; and

• Focus group guides for discussions with health workers to qualitatively capture their perceptions of the various recruitment and retention strategies.

The instruments were drafted and pilot-tested with a purposive sample of health workers in a different health district, Chongwe. Minor revisions were then made to the instruments and research ethics approval obtained from the research ethics boards at the University of Zambia and Dalhousie University. The administration of surveys and data collection was carried out directly by members of the research team. Data were collected from health workers in October 2010 at one rural health centre, one remote health centre, and the district hospital in both Gwembe and Chibombo. An interviewer from the research team provided questionnaires to the district managers, provincial health offices, and Ministry of Health at that time to fill in the administrative data and return to the research team either in-person, via mail, or via-email, depending on the facility.

Key themes emerging from focus group discussions were identified from transcripts using thematic analysis [15]. Descriptive analyses of quantitative data were conducted. Job satisfaction was derived from the sums of responses to a series of 22 questions on the health worker questionnaire, each with 5-point Likert scale responses, asking respondents to indicate their level of satisfaction with various aspects of their jobs. Response options to the individual questions ranged from 1 (very dissatisfied) to 5 (very satisfied); thus the possible total job satisfaction scores range from 22 to 110. To ease interpretation of results, individual scores were converted to percentages, making the lowest possible job satisfaction score 0, and the highest possible was 100.

The remaining key outcome variables were categorical. Respondents were asked how often they consider leaving their jobs, with response options of never, seldom, often, and all the time. Respondents were also asked how likely it was that they would leave their position in the coming year; possible responses were not at all likely, not very likely, fairly likely, and very likely. Finally, respondents were asked which of the initiatives in which they had participated was the most effective in encouraging them to remain in their jobs.

Relationships at the individual level between health worker participation in each recruitment and retention scheme and job satisfaction, likelihood of leaving, and frequency of considering leaving their jobs were investigated using regression models [16]. A linear regression model was used for the continuous job satisfaction variable. Health workers’ self-reported likelihood of leaving their jobs was modelled using a binomial logistic regression model, with the variable dichotomized into not at all or not very likely vs. fairly or very likely. The un-dichotomized variable—i.e. with all four response categories—was also modelled using multivariate logistic regression. The frequency with which health workers reported considering leaving their jobs was modelled using an ordered logistic regression model, given the inherent ordering of the possible responses. Dummy variables for respondents’ age group, gender, district, and profession were used as controls. Starting with all possible variables in the models, a backward elimination process was used to optimize each model’s fit by removing control variables that did not contribute to the model’s predictive power.

Participation in the child education component of the ZHWRS was not included as a variable in the regression analysis as no respondents reported participating in it; it was later discovered that this component of the ZHWRS had been discontinued. Participation variables for the graduate recruitment allowance and the on-call allowance were also excluded as they were perfectly collinear; only respondents who participated in one participated in the other. In addition, there was considerable correlation between respondent education and profession (e.g. only doctors and pharmacists had degrees), therefore education was excluded from the modelling. This left seventeen retention and recruitment schemes whose participation rates were tested for relationships with each of the three outcome variables, i.e. 51 different regression models were created.

## Results

### Descriptive analysis

Forty-five health workers, representing most of the cadre of health care workers working at the facilities sampled, and approximately 25% of all 180 health workers in both districts, completed the questionnaires in an interview setting and participated in focus group discussions. As shown in Table 1, most respondents (67%) were aged between 20 and 40 years, and most (75%) held a professional diploma or certification as their highest level of formal education. Males and females, and workers from the two districts, had roughly equal representation at close to 50% each. Nurses were the most common profession in the sample, accounting for 40% of respondents, followed by midwives at 14%. (Table 1)

**Table 1:** Sample Characteristics

Attribute		Breakdown (%)
Age	<20 20-29 30-39 40-49 50-59 60+	0 38 29 18 13 0

Gender	Female Male	51 49

Profession	Clinical Officer Doctor EHT Midwife Nurse Pharmacist Other	11 7 9 14 40 1 13

Education	Secondary school only Professional certificate/diploma Bachelor’s degree Missing	11 75 9 4

District	Chibombo Gwembe	49 51

EHT: Environmental Health Technologist

A total of nineteen health worker recruitment and retention strategies were found to have been implemented in Chibombo and Gwembe. One of these—the ZHWRS—has five distinct components, which were treated separately for the purposes of this analysis. The various initiatives and their participation rates in the districts are shown in Table 2.

**Table 2:** Retention and Recruitment Initiative Sample Participation Rates

Initiative	Sample Participation Rate (%)
Rural hardship allowance Uniform maintenance allowance Commuted overtime allowance Night duty allowance Housing allowance Transportation equipment (motorcycle) Building electrification (solar panels) Building water provision (bore hole/well) Radio equipment ZHWRS salary top-up ZHWRS housing rehabilitation ZHWRS professional development priority Remote hardship allowance Doctors’ on-call allowance CHAZ top-up Rural professional development priority Graduate retention allowance ZHWRS car loan ZHWRS child education allowance	80 80 69 62 60 55 53 51 40 22 22 18 13 7 7 7 2 2 0

#### Initiative participation

There was substantial variation in the level of participation from scheme to scheme. Those with the highest participation were the rural hardship allowance (80%), the uniform maintenance allowance (80%), and the commuted overtime allowance (69%). The strategies with the lowest participation were the child education and car loan components of the ZHWRS, the graduate retention allowance, and the rural professional development priority, with fewer than 5% of respondents indicating that they had participated in these strategies. The most common reasons cited for not participating in strategies was lack of awareness of them, although some are available only to certain cadres, such as doctors (e.g. on-call allowance).

#### Job satisfaction

Respondents’ total job satisfaction scores ranged from a low of 24% to a high of 92% with a mean 61% and a median of 64%. The mean being lower than the median and the greater difference between the 25^th^ percentile and the median compared to the 75^th^ percentile and the median show the data are slightly skewed toward lower responses; however there were no extremely low scores.

#### Self-reported frequency of considering leaving one’s job

Only eighteen percent of respondents reported they never consider leaving their jobs. Twenty seven percent reported they seldom do so, while 39% reported they do so often, and 16% said they do so all the time. Dichotomizing into ‘seldom or never’ versus ‘often or all the time’, then, creates groups of 45% and 55% of the sample, respectively.

#### Self-reported likelihood of leaving job

Only twenty-seven percent of respondents reported that it was not at all likely they would leave their jobs in the coming year. Twenty percent said it was not very likely, 30% said it was fairly likely, and 23% said it was very likely.

#### Most effective initiatives

Respondents were asked which of the nineteen recruitment and retention strategies identified was the most effective for them personally. The majority of respondents identified the rural hardship allowance as the most effective initiative; no more than 5% of respondents identified any other initiative as most effective.

#### Turnover

The district managers’ questionnaires indicate that there was substantial turnover in the health workforces in Chibombo and Gwembe over the five-year period from 2005-2009, with annual losses ranging from 2% to 16% of the professional health workforce in each district. These losses were partially mitigated by inflows of new health workers; over this period, there was a 3% net gain of professional health workers in Chibombo and an 8% loss in Gwembe. The majority of staff losses were reported as being due to deaths, retirements, and redirections (staff moves directed by administration). However, both districts report being significantly understaffed, with many unfilled positions.

### Regression analysis

Comparisons of confidence intervals for the mean job satisfaction scores for different groups of respondents found no significant differences in job satisfaction between respondents of different age groups, genders, professions, levels of education, or districts.

As noted above, 51 regression models were created. In each of these models, the control variables were more significant predictors of the outcome variables than any of the participation variables. In the interests of brevity, the modelling results for only one scheme per outcome variable – the one with the lowest p-value – are presented in detail.

The scheme with the lowest p-value in predicting job satisfaction scores through a linear regression model was the uniform maintenance allowance. This model had an F-statistic of 2.225 and a p-value of 0.033, indicating that it explains a significant portion of the variance in job satisfaction scores among these respondents. The model’s coefficients and their associated p-values are shown in Table 3.

**Table 3:** Model Coefficients. Job Satisfaction Score by Uniform Maintenance Allowance Participation

Variable	β	***t***	p-value
(Constant)		4.157	.000
Uniform Maintenance Allowance Participation (Yes vs. No)	-.237	-1.417	.166
Gender (Female vs. Male)	-.462	-2.113	.042
District (Gwembe vs. Chibombo)	.469	2.831	.008
Profession = Doctor (vs. Nurse)	-.337	-1.868	.071
Profession = Midwife (vs. Nurse)	-.312	-1.763	.087
Profession = Clinic Officer (vs. Nurse)	-.242	-1.373	.179
Profession = EHT (vs. Nurse)	-.249	-1.390	.174
Profession = Pharmacist (vs. Nurse)	.013	.089	.930
Profession = Other (vs. Nurse)	-.236	-1.475	.150
Age = 30-39 (vs. 20-29)	.212	1.253	.219
Age = 40-49 (vs. 20-29)	.244	1.438	.160
Age = 50-59 (vs. 20-29)	.516	3.223	.003

Respondent gender, district, and being aged 50-59 are all significant at the α=0.05 level, indicating that female respondents tended to report lower satisfaction than males, health workers in Gwembe tended to report higher satisfaction scores than those in Chibombo, and respondents aged 50-59 reported job satisfaction scores 23% higher than those aged 20-29. In addition, doctors and midwives reported 20% and 13% lower satisfaction scores than nurses; these coefficients were significant at the α=0.1 level. Participation in the uniform maintenance allowance, however, was not a significant predictor in the model.

The scheme whose participation had the lowest p-value in predicting health workers’ dichotomized self-reported likelihood of leaving their jobs, through a binomial logistic regression model, was the housing allowance. The model achieves 75% concordance between predicted and observed values, and its likelihood ratio has a p-value of 0.09, meaning that at the α=0.1 level we can reject the hypothesis that the model predicts respondents’ likelihood of leaving their jobs no better than chance or an ‘empty’ model. The coefficients, odds ratios, and associated p-values of the model components are shown in Table 4.

**Table 4:** Model Coefficients. Likelihood of Leaving Job by Housing Allowance Participation

Variable	Coefficient	Odds Ratio	p-value
Housing Allowance Participation (Yes vs. No) District (Gwembe vs. Chibombo) Age = 30-39 (vs. 20-29) Age = 50-59 (vs. 20-29) Gender (Female vs. Male) Profession = Clinical Officer (vs. Nurse)	1.02 1.26 1.35 1.23 1.18 -0.04	2.77 3.53 3.87 3.42 3.27 0.96	0.16 0.12 0.12 0.28 0.10 0.98

None of the model components meets the α=0.05 level of significance, although several are at or near the α=0.1 level of significance. According to the model, participants in the housing allowance scheme were about 2.8 times as likely as non-participants to report that it was fairly or very likely that they would leave their jobs in the coming year. Respondents in Gwembe were about 3.5 times more likely to report that they would likely leave their jobs in the coming year. Respondents aged 30-39 and 50-59 were about 3.4 and 3.3 times as likely as those aged 20-29 to report that they would likely leave their job in the coming year. Females were about 3.3 times as likely as males to report that they would likely leave their job in the coming year. Finally, clinical officers were about 4% less likely than nurses to report that they would likely leave their jobs in the coming year.

The scheme in which participation was the best predictor of respondents’ self-reported frequency of considering leaving their jobs, using an ordered logistic regression model, was the housing allowance. The model achieves 78% concordance and has a likelihood ratio with a p-value of 0.02, suggesting that this collection of independent variables is a significant predictor of the frequency with which respondents consider leaving their jobs. The model coefficients, odds ratios, and associated p-values are shown in Table 5.

**Table 5:** Model Coefficients. Frequency of Considering Leaving Job by Housing Allowance Participation

Variable	Coefficient	Odds Ratio	p-value
Housing Allowance Participation (Yes vs. No) District (Gwembe vs. Chibombo) Age = 30-39 (vs. 20-29) Age = 40-49 (vs. 20-29) Age = 50-59 (vs. 20-29) Gender (Female vs. Male) Profession = Clinical Officer (vs. Nurse) Profession = Doctor (vs. Nurse) Profession = Environmental Tech (vs. Nurse) Profession = Midwife (vs. Nurse) Profession = Pharmacist (vs. Nurse)	0.73 1.19 0.87 -2.13 1.16 1.61 0.86 0.39 1.56 1.42 2.83	2.08 3.30 2.39 0.12 3.18 4.99 2.38 1.48 4.75 4.12 16.87	0.29 0.13 0.33 0.06 0.28 0.10 0.53 0.81 0.16 0.22 0.21

None of the variables in the model is significant in predicting the frequency with which respondents report considering leaving their jobs at the α=0.05 level of significance, although, as in the previous model, several are at or near the α=0.1 level. The model parameters indicate that participants in the housing allowance scheme tended to consider leaving their jobs more often than non-participants; more specifically, that respondents who participated in this scheme were about twice as likely as those who did not participate to be in a more frequent category of considering leaving their job. The parameters also indicate that respondents in Gwembe tended to consider leaving their jobs more often than respondents in Chibombo, that respondents aged 30-39 and 50-59 considered leaving their jobs more often than those aged 20-29, while respondents aged 40-49 tended to do so less often than the 20-29-year-olds. Females considered leaving their jobs more often than males, and respondents of every other profession considered leaving their jobs more often than nurses.

### Qualitative analysis

The main themes emerging from focus group discussions with the health workers in both districts were that they are not satisfied with their living and working conditions and that the currently available recruitment and retention strategies are not adequate to address these problems. Housing, road conditions and access to communications and utilities were described as being poor, and health workers reported little access to educational opportunities for their children. A number of problems with working conditions were cited, including chronic shortages of staff, supplies, and equipment, and unhygienic facilities.

Consistent with the quantitative data, participants identified the rural hardship allowance as the scheme that does the most to keep them in their jobs; however they reported that it was insufficient in its amount. This criticism was applied to all the financial allowances as part of the strategies, as they seemed not to reflect current costs of living. Examples cited were that the amounts for the ZHWRS car loan were insufficient to purchase the kind of vehicle needed to navigate rural and remote roadways, and that the rural hardship allowance is less than the cost of transport back and forth from the bank (necessary to collect wages)^ii^. Professional development opportunities were perceived positively by those who had accessed them, however it was reported that these are very seldom accessible to health workers in rural and remote areas.

Also consistent with the quantitative data, participants reported a lack of understanding of several of the strategies, which they attributed to poor communication about them from an organizational level. They also perceived that the strategies were implemented inconsistently and that the criteria for eligibility were not clear to them.

Participants reported that the main reasons they remained in their jobs were local family attachments or pending retirement as opposed to any satisfaction with the jobs themselves. None of the recruitment and retention strategies were described as satisfactory.

## Discussion

Quantitative analysis shows little or no relationship between participation in the various recruitment and retention strategies for health workers and a) job satisfaction, b) intention to stay in their jobs, or c) the frequency with which those health workers consider leaving their jobs. Characteristics of individual health workers—such as their age, gender, profession, and district—appear to have more to do with their intention to stay in their jobs and the frequency with which they contemplate leaving them. The facts that the main reasons for turnover of health workers in these districts seem to be deaths and retirements as opposed to resignations or requests for transfers, and that most recent losses have been replaced, suggest that recruitment and retention in these districts are not without some success. However, with a sample of only two districts it is not possible to determine what portion of that success is attributable to specific strategies. Further, the fact that both districts are still very understaffed indicates there remains considerable room for improvement in recruitment and retention activities.

Consistent with these findings, qualitative analysis indicates that the most significant issues impacting health workers in these districts are related to their living and working conditions and are not addressed by existing recruitment and retention strategies. Further, data indicate that these strategies are not considered satisfactory or well understood by many health workers. These findings, consistent with other research on retention of health workers (HWs) [17,18], indicate the need for continued research to better understand the broad range of factors that contribute to HW motivation and retention, and the differing degrees to which different incentives influence their motivation and job satisfaction. Examples of research contibuting to this area include a recent study on HW motivation in rural health facilities in Zambia [19] which showed that gender, profession, type of training, and time in post each influence health workers’ motivation.

This study is limited by the small sample size available for analysis, particularly considering the large number of variables being analyzed. Although highly representative of the facilities surveyed, the generalizability of the results from these respondents to other areas is uncertain. Further, these data include only the perspectives of health workers currently employed in the two districts; those who may have been working in these districts and involved in the various recruitment and retention strategies in the past but have left are not included. The results may therefore be biased toward the perspectives of health workers who have, for any number of reasons, stayed in these districts. A larger sample, data on additional, potentially confounding variables, and data on health workers who have left these districts would all allow for a more rigorous analysis of the impacts—if any—of the various recruitment and retention strategies implemented in rural Zambia. Despite these limitations, when the study findings were presented at meetings of the health workers in each districts, the attendees reported that they were consistent with their experiences.

Based on the findings of the present research, the research team hosted a deliberative forum in Zambia in October 2012. The purpose of the forum was to share the findings with a wide range of stakeholders, and to acquire stakeholder consensus and to authenticate the recommendations that were made through a consultative process ^iii^. Stakeholders in attendance included representatives from government (including the Ministry of Health, the Ministry of Community Development and Maternal and Child Health, and the Prime Minister’s Office), NGOs (e.g., Clinton Health Access Initiative), the World Health Organization, funders (IDRC), HWs, professional groups (e.g., Health Professions Council of Zambia, Zambia General Nursing Council), educational institutions (e.g., Lusaka School of Midwifery, Lusaka School of Nursing, and Lusaka Apex Medical University) and researchers. The recommendations developed are aimed at the Ministry of Health to inform not only the work within their own portfolio, but also their collaboration with other areas of government, including the Ministry of Community Development, Mother and Child Health, the Ministry of Finance, the Cabinet Office, and others.

The recommendations were largely focused on developing and implementing strategies for recruitment and retention that are more aligned with and reflective of the realities of living and working in rural and remote areas. Examples of potential strategies participants suggested for exploration included:

• A multi-layered orientation processes for newly hired health workers to help them better understand the context, expectations and procedures of the facility and district to which they are posted;

• Decentralizing HRH recruitment and hiring to provincial and district levels, providing districts with the authority to allocate resources to facilitate task shifting for the provision of necessary services when trained health workers are not available;

• Improving and strengthening existing communication forums and other mechanisms to enhance communication among different levels of administration, health care providers, and communities regarding HRH recruitment, promotions, and deployment;

• Redefining the classification of districts and facilities as rural, remote, etc.;

• Increasing collaboration between government ministries, NGOs, and the private sector could help facilitate improved infrastructure in these areas such that living and working conditions are improved;

• Revising the quantum of the financial incentives on an on-going basis to reflect changes in inflation, cost of living, and ensure that they are appropriate across cadres to duration of training, experience, and workload/hours worked.

## Conclusion

Results of the quantitative and qualitative analyses suggest there is little association between health worker recruitment and retention strategies in place in Gwembe and Chibombo and health worker recruitment and retention. What relationships may exist seem to be less important to health worker recruitment and retention than individual characteristics of health care workers themselves as well as their living and working conditions, many of which are outside the scope of the recruitment and retention strategies. This suggests that even if existing strategies are reviewed and adjusted—for example, if financial allowances are increased on par with costs of living—efforts to retain and recruit health workers in these areas may be more effective if things like housing, communications, road conditions, and educational opportunities are addressed. In the shorter term, although the government of Zambia has already begun working to make practical improvements to existing schemes (such as updating the amounts of allowances and other financial incentives), there remains room to improve others. For example, additional planning could help to make professional development opportunities – which were rated positively by those who were able to participate in them – more accessible to health workers in rural districts. In the long term, because the challenges facing Zambia’s health sector have causes – and implications – across other portfolios, a multi-faceted, intersectoral response is required to address these.

## List of abbreviations

CHAZ: Churches Health Association of Zambia; DFATD: Foreign Affairs, Trade and Development Canada; GHRI: Global Health Research Initiative; HRH: human resources for health; HW: health worker; IDRC: International Development Research Centre; MoH: Ministry of Health; NGO: Non-governmental organization; ZHWRS: Zambia Health Workers Retention Schemes

## Competing interests

The authors have no competing interests to declare.

## Authors' contributions

FMG, GTM, AM, and SN led the conceptualization, data collection, interpretation of findings, writing and editing. ML led the engagement and communication with key stakeholders and decision-makers from MoH throughout the project and contributed to interpretation of findings, writing and editing. CMM contributed to the interpretation of findings, writing, and editing. AG contributed to data collection, interpretation of findings, writing, and editing. JR contributed to writing and editing. All authors have read and approved the final manuscript.

## Declarations

The publication costs associated with this article are funded by Foreign Affairs, Trade and Development Canada and the International Development Research Centre through the Global Health Research Initiative.

This article has been published as part of *Human Resources for Health* Volume 12 Supplement 1, 2014: Addressing the human resources for health crisis through task-shifting and retention: results from the Africa Health Systems Initiative's research component. The full contents of the supplement are available online at http://www.human-resources-health.com/supplements/12/S1

## Endnotes

i. An additional component of the study focused on identifying critical health service needs in these districts and comparing them with those that can be provided by the existing workforce; this component of the study is not discussed in this paper.

ii. At a deliberative forum with key stakeholders in Zambia in October 2012, the research team learned that several updates had been made to the strategies since the time of project analysis. For example, child education allowance and commuted overtime allowance were eliminated; the process for obtaining car loans was streamlined to eliminate redundancies; and there had been discussions about expanding on-call allowance to additional cadres.

iii. The recommendations developed at the deliberative forum were based on the combination of findings from the dual components of the project: 1) the evaluation of retention and recruitment schemes, and 2) the service-based assessment of the alignment of between critical health service needs in the districts compared to those that can be provided by the existing workforce. The recommendations presented in this paper are those most related to the first component, however, it should be noted they have been informed by findings from the second component.
